# Weighing up the risks and benefits of community gambling venues as recreational spaces for people with lifelong disability

**DOI:** 10.1186/s12889-020-08654-0

**Published:** 2020-06-12

**Authors:** Hannah Pitt, Samantha L. Thomas, Joanne Watson, Russell Shuttleworth, Kevin Murfitt, Susan Balandin

**Affiliations:** 1 Institute for Health Transformation, School of Health and Social Development, Faculty of Health, Deakin University, Geelong, Australia; 2 Department of Anthropology, Goldsmiths, University of London, London, UK

**Keywords:** Gambling, Intellectual disability, Gambling venues, Recreation, Leisure

## Abstract

**Background:**

Community gambling venues (pubs and clubs) are commonly associated with leisure and recreational options in Australian communities. While these venues offer a range of activities and facilities, including social opportunities, sporting facilities, live entertainment, they also contain gambling products that are known to cause significant harm to individuals, their families and communities. Although researchers have explored how adults and children engage with these venues, there is limited understanding about the potential risks and benefits of these venues for people with lifelong disability.

**Methods:**

Semi structured interviews were conducted with nineteen people aged 20–70 years with lifelong disability (includes in this context intellectual disability, autism spectrum disorder, ADHD, and learning disability), predominately intellectual disability. The interviews occurred in a large Australian city and explored interviewees’ experiences and attitudes towards pubs and clubs. Using a range of visual prompts (if needed), participants were asked to describe their engagement in different activities offered within the venue. Interviews were audio-recorded and transcribed, with a thematic analysis used to identify themes across the group.

**Results:**

Most participants attended venues with family, friends, and supporters, with a few attending on their own. Participants described socialising in the venue, going for reduced price meals, and attended for a range of activities including recreational activities, live entertainment and sport. Some participants also valued being a member of venues, and the interactions with staff members. While participants were cautious about the consumption of alcohol, most had gambled, particularly on electronic gambling machines (EGMs, pokies, or slots). Some participants stated that they had experienced problems with gambling.

**Conclusions:**

While many people with lifelong disability have positive experiences in pubs and clubs, some are vulnerable to the harms associated with risky products such as gambling within the venue. While it is important to acknowledge the positives associated with recreational facilities and encourage engagement in leisure activities for people with lifelong disability, further consideration is needed to ensure people are informed and protected from the harms associated with gambling and other products that are provided within these spaces.

## Background

### People with lifelong disability and leisure and recreational activities

The contribution of engagement in leisure and recreational activities to enhancing quality of life and well-being, is well documented [1, 2]. Article 30 of The United Nations Convention on the Rights of Persons with Disability, to which Australia is a signatory, affirms the right of people with disability to be provided with equal recreation and leisure within mainstream society [3]. Despite this social and legislative backdrop, people with lifelong disability participate significantly less in leisure activities than their non-disabled peers [4]. Lifelong disability for the purpose of this study includes intellectual disability, autism spectrum disorder, ADHD, and learning disability. While lifelong disability could also include people who have had a physical disability since birth, for the purpose of this study this group was not included as it was assumed they would have a different risk profile.

Engagement in leisure and recreational activities may have positive implications on increasing social inclusion for people with lifelong disability. Simplican et al. [2015] defined social inclusion as “*the interaction between two major life domains: interpersonal relationships and community participation*” ([5], p. 18). This study described the benefits of social inclusion as increasing an individual’s potential to build friendships, increase community involvement, along with having a direct impact on the individual’s happiness, confidence and mental health [5]. Abbott and McConkey [2006] explored the facilitators and barriers to social inclusion from the perspective of people with intellectual disability [6]. They found that people with intellectual disability perceived a lack of knowledge and skills in identifying opportunities, and a lack of confidence in accessing facilities as barriers to social inclusion. Additionally, participants in Abbott and McConkey’s [2006] study identified, the location and accessibility to venues, staff restrictions, limited activities available, and experiences of negative attitudes from the community, as barriers to social inclusion [6]. Despite clubs and pubs being accessible and popular leisure and recreation venues for many Australians, little is known about how people with lifelong disability utilise these venues or if they use the gambling facilities available.

### Community gambling venues in Australia

Pubs and clubs are the two main types of community gambling venues in Australia. The main difference between pubs and clubs is that pubs are for profit businesses and clubs are member based not for profit organisations (and include Returned and Services League (RSL), social, sporting, cultural, and workers clubs). Pubs and clubs usually contain a range of non-gambling activities such as moderately priced food and drinks, social activities, sporting facilities, and live entertainment [7]. However, they also contain potentially harmful gambling products ranging from raffles and bingo to more risky products including sports betting facilities, and electronic gambling machines (EGMs) [8], which are commonly known as pokies or poker machines (Australia), fruit machines (UK) or slot machines (US). The activities that are within clubs are considered affordable, this is because they are subsidised from the revenue made from gambling products, particularly EGMs. This means there is a tension between the co-location of recreational spaces that encourage community engagement and the access to potentially harmful products such as gambling. This is especially pertinent in Victoria, Australia where there are 495 venues (260 pubs and 235 clubs) containing 26,466 EGMs which comprises over 90% of the state’s allocated number of EGM licences [9]. EGM expenditure in Victoria was $2.7 billion in 2017/18 [9]. There were almost 7 million (39%) regular (monthly) gamblers in Australia in 2015, and 21% of those regular gamblers gambled on EGMs [10]. This is concerning given that research has found between 40 and 60% of EGM users will experience harms associated with gambling [10, 11]. Harms associated with gambling are not only limited to monetary issues such as financial instability or bankruptcy but also include a range of other negative social and health consequences. These include impacts on mental health (i.e. anxiety and depression), substance use, relationship breakdown, homelessness, suicidal ideation, family violence and crime [12–16]. Given this data, it is important to understand the experiences of people, including those with lifelong disability, who attend these venues. In particular, to explore the reasons why they attend, the activities they participate in while they are there, and whether they are at risk of harm from gambling.

Researchers have begun to explore the reasons why population sub groups such as families, older adults and people who gamble interact with venues [17–19]. Studies have demonstrated that the majority of participants attend venues for non-gambling reasons, including restaurants, social interactions, recreational facilities, and perceive that venues are safe, and have a friendly or inviting atmosphere [17–19]. Bestman and colleagues (2018) conducted an online survey with 500 participants in New South Wales aged 16–82 years, and found that many reported that they attended the venue because of the affordability of food and drinks and the provision of services including entertainment and to attend functions such as sports presentation nights, or family celebrations [17]. Research with 126 older adults in Victoria has demonstrated that community gambling venues are used because of the reasonable cost of food, drinks and activities, the accessibility of venues within the local community, the availability of the courtesy bus to ensure free and convenient transport, the opportunity to reduce feelings of social isolation and loneliness and build social connectedness [20]. Participants also noted that these venues were spaces where people could feel comfortable to sit for extended periods of time without feeling the pressure to be moved along [20]. Studies have also identified that a driver of the use of community gambling venues is their structural accessibility, including accessible parking, and disability friendly facilities and activities [18].

There are significant positive factors associated with venues, particularly with community clubs for the health and wellbeing of people who may otherwise feel excluded in communities. However there are also serious concerns relating to the presence of gambling activities within these spaces. For example, evidence suggests that those who regularly use community gambling venues for non-gambling activities also more regularly use gambling products within these venues. Bestman and colleagues (2018), found that the more often people attended venues the more likely they were to gamble on EGMs, and the more likely they were to experience some form of gambling harm [17]. Due to the negative consequences associated with gambling it is important to understand how subgroups who may be marginalised or vulnerable within community settings, may use community gambling venues, and the potential risks and benefits associated with regularly interacting with these venues.

### Community gambling venues and people with lifelong disability

There is limited research that documents how people with lifelong disabilities may use community gambling venues. There is some evidence from industry documents that suggests that community gambling venues view themselves as a supportive and inclusive space for people with disabilities. For example, the peak bodies for clubs, Clubs Australia and Clubs New South Wales, describe their venues “*as community hubs”* that are *“well placed to support the needs of people with disability and promote inclusiveness to their members, guests and staff*.” ([21], p. 6). There has been a small amount of international research that can be drawn on to understand the reasons why people with lifelong disability may go to pubs or the benefits they receive from attending pubs. Researchers have suggested that there are a number of reasons why people with a disability may find gambling venues appealing, including the accessibility of the venue, the range of activities within it, a chance to escape, to socialise in places they feel safe and included, and for fun and excitement [22]. Taggart et al. [2007] interviewed ten people with an intellectual disability in the UK. Although the aim of the study was to explore the pathways into substance abuse problems, predominately alcohol, the results also highlighted the initial reasons why people with intellectual disability attended pubs [23]. The researchers found that they often drank alcohol or attended bars and clubs to reduce feelings of isolation and loneliness, and to increase social interactions and fit in with society [23]. Many participants reported that they had limited recreational opportunities, and used these venues as a way of meeting friends or enjoyed having people around them. Another study by Forrester-Jones and colleagues (2006) explored the social connections of 213 people with intellectual disability [24]. This study found that owners and staff of pubs, cafes and shops were some of the few connections that people had in their lives that were not associated with services [24].

### Approach

This study took a participatory approach. An advisory group made up of six people with lifelong disability from the researchers’ networks and who had expressed interest in this project guided the development of the study. Two meetings with this advisory group involved discussing the key aims of the project, and reviewing the interview schedule to identify if the wording and language was appropriate and well understood. Working with an advisory group helped ensure that the research was inclusive and relevant to people with lifelong disability and shape the research questions and themes for investigation in a way that was meaningful to people with lifelong disability. Given the limited qualitative research in this area, the aim of this study was to understand the role of pubs and clubs in the lives of people with lifelong disability. The study was guided by three research questions:

1. What are the experiences and attitudes of people with lifelong disability who attend pubs and clubs?
2. What are the activities that they participate in while at pubs and clubs?
3. What is the level of awareness of, and engagement with, gambling products within these venues?

## Methods

### Sampling and recruitment

The researchers approached a disability advocacy organisation, to facilitate the recruitment and sampling for this project. The researchers attended a meeting at the organisation to describe the project to potential participants, including the expectations and requirements of the study, and the inclusion criteria. Individuals were included if they had visited a community gambling venue in the last year, and were able to consent to their own participation. Participants were required to be over 18 years of age, and were living in Victoria. As all recruitment was conducted through this organisation, it was assumed that all participants who were involved had lifelong disability, predominately intellectual disability, given the nature of the services this organisation provided. Based on advice from disability researchers and the organisation there was no need to test or confirm the nature of the disability from the participants. Deakin University Human Research Ethics Committee gave ethical approval for the study. All participants received a $50 grocery voucher for their participation.

### Data collection

Semi-structured interviews occurred over four data collection time points from July to September 2018. Interviews lasted between 10 and 40 min, depending on each participant’s level of understanding and desire to discuss their experiences. The interviews were audio recorded with permission. One interview was conducted with two participants at the same time, throughout the interview each question was asked of each participant individually.

The interview schedule involved four sections. The first included general demographic questions such as age, gender, residential suburb, and living arrangements. The second comprised the experiences of the venue, including the frequency of attendance, accessibility of the venue, engagement in the venue, and attitudes towards the venue. The third contained knowledge and attitudes towards gambling, including product use and knowledge, and awareness of harm minimisation strategies. The fourth explored potential strategies for addressing the harms associated with gambling within venues. The interview schedule can be found as an additional file.

Two picture boards were used to facilitate discussions with participants. This technique has been used in studies with young people to explore gambling and gambling related themes [25–27], including the intended use of activities within clubs [25]. This technique has also been used in research involving people who may have difficulties accessing written information alone [28, 29]. The first picture board contained a number of different gambling and non-gambling activities that people may have seen or participated in within pubs or clubs. The second picture board included gambling products such as EGMs, sports betting, horse betting, keno, and raffles. Researchers also had pictures of each individual gambling product on a separate piece of paper in case the picture board was overwhelming or if the interviewer needed to focus discussion on a specific product.

### Data analysis

Interviews were transcribed by a professional transcription company and were uploaded to QSR NVivo 12 to assist with the management of the data. A thematic analysis was conducted whereby transcripts were read and re-read to ensure a clear understanding of the content and context of each transcript as a whole and as a group. Initial codes were created, which then were used to develop broader themes [30]. Interpretation of the results and findings were discussed within the research team throughout data collection and analysis. Themes were reviewed and the final presentation of results were constantly checked to ensure they were encompassing a true representation of data. Quotations from the data were used to illustrate different thoughts and opinions and have been corrected for grammatical errors.

## Results

There were 19 participants and 18 interviews conducted in this study. The age of participants ranged from 20 to 70 years old with an average age of 44 years. Just over half of the sample were male (*n* = 10, 52.6%).

### Attendance at community gambling venues

All participants were familiar with pubs and/or clubs, with many participants able to name at least one specific community venue that included gambling that they had either attended or were aware of. However, participants had difficulty recalling the frequency of their attendance. Some participants had trouble specifically quantifying how often they went and used terms such as “*a lot*”, “*sometimes*”, or “*not very often*”. Other participants could conceptualise how frequently they went to these venues by describing key events for which they attended, including exercise classes, birthdays, sporting event, and annual events such as a war memorial day. Others were more specific about their attendance, with answers demonstrating that attending these venues were part of weekly routines. For example, one woman said she went every Saturday, and another woman used her routine as a measure of how frequently she went to the RSL with her husband:

*We do it like once a fortnight maybe. We go on, on my payday, we will go that’s our social get out together.* – Female.

Although each participant had different attendance experiences, the venue they attended often influenced how often they went and who they went with. For example, some participants described family members, partners, friends, or support workers taking them to venues, with only a few stating that they went independently to a venue either by themselves or with friends. Participants who went by themselves mostly went to those venues that they were able to access by walking, stating that they went to venues that were easy to get to and close to where they lived. While most participants went to venues in their local neighbourhood, one participant went to a pub that he had previously lived near to in order to meet up with friends. While he had to take two trains to get to the venue, he considered it to be “*a nice trek*” and that it gave him “*something to do*”. Another, described experiencing harm from gambling, recounted how she had previously attended an RSL by arranging a taxi to pick her up: “*10 dollars there, 10 dollars back*”. She also recalled that she felt this was the “*safest place*” for her to go. However, this participant described that after experiencing problems with gambling in the venue, she now chose to go to a pub closer to her house that did not contain gambling products.

Age played a role in decision making about which venue to attend. For example, older participants preferred going to RSLs or clubs, as they perceived that they were “*friendlier than the hotels*”. One participant described going to a few different venues with friends to access different things that they liked in different venues:

*… we like to go to* [name of venue 1]*, because of the chef, he is really good, so we go for meal, but if we don’t want to have a meal, we go to* [name of venue 2]. – Female.

Another participant described attending a particular venue because of the different activities it provided for her family, including the children, when they were there. The range of activities and the perception that it was family friendly made it a more attractive environment than other community locations. This participant described how her family would take turns at either gambling or watching the children:

*So if we go watch* [her cousins] *play football, we can watch them play football and then have lunch. And the kids can play. One line of people watch the kids and then we do a swap around …*. S*o, some will go play the pokies, some will keep an eye on the kids and then we’ll swap*. – Female.

Some participants described that disability access was a positive feature of the venue that influenced their choice to attend. One participant said there would not be anything they would change about the venue and that the venue and staff had been “*quite responsive*” when requests about disability access were raised. While participants who spoke about disability access were predominately positive, they did recognise that access can often be problematic, particularly for those using wheelchairs and accessing bathrooms, and offered potential areas of improvement. For example, a participant said, “*sometimes I don’t agree with how big the disabled toilets are*”. Another participant who attended the venue frequently described how she wanted to be able to use the courtesy bus but it did not have wheelchair access. A few participants also talked about wheelchair access to the front door of the venue, with one participant describing how the venue could ensure it was more inclusive:

*Some are friendly* [for people with disability] *but some are, they need to look at the building and say what can we fix, like the front doors? You can’t get a wheelchair in there. Because you have to open the door and then hold it with your leg and then push the wheelchair in. So they need to look at things like that.* – Female.

### Awareness and use of activities within pubs and clubs

Participants described socialising within pubs and clubs as one of the main outcomes of going to the venue:

*We go there because it’s a nice place, we also catch up with people, friends there, we have a drink there, we socialise*. – Female.

A few participants indicated that they enjoyed talking to people who they did not know while in the venue. Some participants described socialising within the gambling spaces in the venue. For example, one participant described socialising with other people while in the “*pokie room*”, when celebrating winning money. Others stated that while they rarely talked to other people, they enjoyed going somewhere where they felt they were in a social environment, could attend by themselves, and felt safe and included.

Participants also described the dining and entertainment options in the venue, with most stating that while they were there they had either a lunch or dinner. For many participants the price of meals available at venues was a key influencer in their decision to attend rather than dining elsewhere. Being a member of these venues led to even cheaper meals:

*It is cheaper at the RSL because if you go to any other restaurant for what we have because I am gluten free. You are paying a lot more. You can pay up to $40 for a meal where I will be paying because we are members of the RSL and we get members price.* – Female.

Several participants acknowledged that there was a bar in the venues they attended; but only a few stated that they consumed alcohol. For example, one participant would go to the pub for a “*couple of drinks*”, with one of the incentives being that the beer was cheap to buy at this pub, while another participant said he went to pubs or clubs to “*get drunk*”. This particular participant described a situation where he had won while gambling on EGMs, and used that money to buy alcohol:

*I took the money out, bought a beer, bought a lady a beer, we exchanged a few cigarettes and had a bit of a chat*. – Male.

Participants also recalled, after being prompted with the picture board, that they watched sport, or the live music or entertainment that was offered. For example, one participant recalled *“Oh and I can’t forget I love the live music as well, and the footy*”. Others took part in a range of health promoting activities - “*exercises there every week and then we have lunch afterwards”.* Some participants described engaging in a range of specific activities, such as music groups. These participants would go to different venues to participate in these groups. For example, the following participant described going to Morning Melodies (a senior citizens event typically consisting of live entertainment and morning tea or lunch):

*I actually have my Morning Melodies every Tuesday...*[Interviewer: So where do you go to morning melodies at?] *Oh at different pubs, I mean not the same place but different places. But then last Tuesday it wasn’t on, there was one on but it was it was too far to travel, that was at the* [name] *RSL. I didn’t go, I wanted to but then it was a bit further out for me to travel that far*. – Male.

Many participants were members of venues, which involved signing up for a membership card where points could be accumulated with any purchases, including gambling, that were made within the venue. The biggest attraction of being a member of a venue was the discounts associated with membership cards, including “*member’s prices*”, and reduced meal prices. The ability to provide discounts for friends and family members was a particularly appealing part of holding a membership card, and enabled participants to invite friends to venues:

*You get discounts on your meals. You can get discounts on your drinks. We have taken our friends, and they get their meals discounted because of us.* – Female.

A few participants knew that points could be earned by spending money on gambling products, and in particular EGMs. One participant was critical of the accumulation and use of membership points for gambling:

*You can choose either points for the pokies or say meals and I chose the meals …* [Interviewer: What do you think about using your points for the pokies?] *I don’t really encourage it, because that’s when people can get into trouble. Yeah, you know that’s why I like to have that choice, to have that choice to have the points off the meals, you know.* – Female.

One participant showed the interviewer four membership cards for different venues, one of which was a silver membership card. This indicated a higher level of membership within a rewards program, usually associated with spending a certain amount of money within the venue. When asked how he was able to receive the silver membership card he said, “*Oh I don’t know you just apply for it, you know the upgrades*”. He also described the benefits of being a member because the venues “*send you out something for your birthday, like if you spend $20 you then get food and a beverage*”.

Participants often described the interactions they had with staff members. A few participants thought the customer service was good, that the staff were “*lovely*”, and were “*really interactive*”*.* One participant recalled the extent of the service that staff members provided:

*They really looked after me. At the hotel if my taxi arrived and I hadn’t quite finished* [using EGMs] *they would come and tell me.* – Female.

Some stated that the staff in the venue knew them by name because they regularly attended the venue. For example, one participant said, “*yeah they know us, because they see us every day”*, while another said “*the staff are nice, they all know us*”. Lastly, another participant, who had very positive attitudes towards the club, indicated that these attitudes were due to how friendly the staff were:

*I mean it is friendly and the staff …, when we walk into the RSL the people at the front desk the people know us by name. It is not just a ‘hi’, it’s “Hi* [participant name] *and* [husband’s name] *how are you?”, and when we go and they say goodbye and they are very friendly. They are very helpful. Like if we ring up to order a table, I don’t even have to say my last name anymore.* – Female.

Finally, participants described a range of gambling products available in the venue. While participants had conservative views about the consumption of alcohol in venues, some regularly participated in gambling. Participants talked about seeing EGMs within pubs and clubs or were aware of the designated gaming room that contains EGMs. For example, one participant described the layout of a club they had been to:

*Yeah on the one side there’s the dining room and one side there’s the pokies room.* – Female.

Keno was another product in the venues recalled by some participants. Although participants were not entirely sure of how Keno worked, they did identify that Keno “*is on the TV at the RSL’s and they’ve got them in the pokies area as well*”. Another participant described Keno by explaining, “*basically you pick numbers and then pretend balls come up*.” Some participants also recalled bingo, although many said bingo was too complicated for them to participate. For example one participants stated “*no I haven’t done bingo because it goes too fast”.* One participant recalled that she had previously gone somewhere to play bingo that had electronic bingo machines, rather than marking off numbers with a pen, which enabled her participation:

*It’s for someone with a disability, you don’t have to mark it at all they* [the machine] *does it automatically, it does it if your number comes up*. – Female.

A few participants talked about participating in raffles when they were in the venue. One participant believed that buying raffle tickets was a way of “*supporting the RSL”.* Some participants described the prizes that were associated with raffles, with meat raffles the most popular. Participants liked raffles because they were relatively inexpensive, there was a chance to win a range of prizes, and it was a way of supporting the club or different community causes:

*With the raffles you can win beer, wine, meat and sometimes because the ladies ancillary do the raffle at the RSL and you put, no pay $5 and you could win a voucher from the ladies ancillary.* – Female.

## Discussion

This study aimed to understand the role of pubs and clubs in the lives of people with lifelong disability. The research had a particular focus on understanding experiences and attitudes towards venues and identifying what activities or facilities people with lifelong disability are aware of and use while at venues. There are three key areas to discuss.

First, this study found that pubs and clubs were venues that people with lifelong disability visited regularly. These venues were perceived as inclusive, affordable, and accessible community spaces, where participants could engage in a variety of activities. Many had memberships with venues, and had formed friendly ‘name basis’ relationships with staff members. Reasons for use of venues were largely similar to those of other marginalised population subgroups, such as older adults, who may find it difficult to access places of inclusion elsewhere in the community [20, 22, 31]. Some participants identified the disability friendly nature of these venues, as exemplified in their description of venues as accessible, affordable and safe, and were spaces that they could attend. It is important to acknowledge the limited recreational spaces that people with disability feel welcome within and to identify the aspects that they enjoy about certain places. However, the inclusive nature of the venues may also create a false sense of security for participants, as while there are many activities that could be beneficial for the health and wellbeing of people with lifelong disabilities, participants also reported that they regularly engaged in high risk activities such as EGM gambling within the venues. The findings from this study could be used as evidence to support the need for a greater range of leisure and recreational opportunities for people to ensure they are able to enjoy their leisure time without the risk of exposure to other harmful products.

Second, participants indicated a range of positive experiences of pubs and clubs when attending with family, partners or friends. Social inclusion and belonging have been identified as important aspects in encouraging positive health and wellbeing, and are often a priority for those working in the disability field [5, 32, 33]. Researchers have found that people with disability attend pubs because they like to be around people, and in some instances perceive that staff are an important part of their social network [24]. For some participants in this study it was clear that venue staff played a large role in building feelings of connectedness and safety within the venue, as some participants described with pleasure that staff knew them by name, and were in some instances the reason why they selected that specific venue over others. Similarities can also be seen with a study by Thomas and colleagues (2020) [20] who found that older adults often attended pubs and clubs because they found the staff friendly and supportive, enjoyed being around people, either through direct social contact or because they liked being in the company of others even if they were not interacting with them [20]. While it is important to acknowledge the different positive social aspects that community gambling venues can provide people with lifelong disability, there are clear risks associated with these aspects of the venues which lead to them regularly attending the venues. Individuals who attend community gambling venues regularly are more likely to also engage in gambling within the venues [17]. This finding calls into question how people with lifelong disability find social networks in community gambling venues, and the reasons why they may not be able to establish these social networks in other community spaces.

Third, gambling products were discussed by participants as something that were present within venues, and which they regularly engaged in. Although most participants had conservative views about alcohol use, they did not seem to hold the same views for gambling products. Whereas only a few participants stated that gambling was the primary reasons for attending venues, many engaged in gambling as a secondary or incidental activity. This is consistent with other research that has found that most people who gamble in venues have originally attended for non-gambling related activities and transition into gambling [17, 18, 20]. This present study highlights the tensions, noted by Bestman and colleagues (2018) [25], between offering community spaces that increase social inclusion and belonging for those who are potentially marginalised or vulnerable within communities, and spaces that may expose them to potentially harmful products. While some participants attended venues by themselves, family members or friends facilitated most attendance. Further research is needed to understand the pathways that encourage attendance into community gambling venues and how people with lifelong disability may transition from non-gambling activities to gambling activities within the venue. This also includes understanding how people with lifelong disability may conceptualise gambling harm, understand the nature of gambling products, and whether they may be additionally vulnerable to experiencing harms associated with these products. This information is particularly important from a consumer protection perspective. If some groups of adults who regularly use gambling venues are not able to clearly understand the risks and harms associated with products, are these products safe to be offered in community based settings? Future research should seek to understand the factors that may exacerbate the risks that people with lifelong disability may experience from regular exposure to gambling products in environments that they perceive as safe and inclusive community spaces.

### Limitations

There were two key limitations to consider. First, participants were recruited from mostly metropolitan locations. It would be interesting to understand how environmental factors such as the accessibility and availability of community gambling venues influence engagement and attitudes towards venues among people with lifelong disability. Further research is needed to understand how location may influence interaction with gambling venues among people with lifelong disability. Second, lifelong disability is a broad inclusion criteria. Therefore it was unclear the exact ‘diagnosis’ for each participant, and while we did not believe that to be important to this study it does mean that the findings are not generalisable to all people with lifelong disability.

## Conclusion

While many people with lifelong disability have positive experiences in pubs and clubs, some are vulnerable to the harms associated with risky products such as gambling within the clubs. While it is important that autonomy in choosing the recreational facilities is encouraged, further consideration is needed to develop strategies to prevent people with lifelong disabilities from developing harms associated with risky products that are provided within these spaces.

## Data Availability

This data will not be made available to ensure the privacy and confidentiality of the participants.

## Abbreviations

ADHD: Attention deficit hyperactivity disorder
EGMs: Electronic gambling machines
RSL: Returned and Services League
UK: United Kingdom
US: United States of America
